# School-Based Participatory Arts for Psychosocial Adjustment and Well-Being in Health Emergencies: An Embedded Mixed-Methods Study

**DOI:** 10.3390/healthcare13212737

**Published:** 2025-10-29

**Authors:** Konstantinos Mastrothanasis, Angelos Gkontelos, Emmanouil Pikoulis, Maria Kladaki, Aikaterini Vasiou, Avra Sidiropoulou, Despoina Papantoniou, Anastasia Pikouli, Evika Karamagioli

**Affiliations:** 1 School of Medicine, National and Kapodistrian University of Athens, 11527 Athens, Greece; kmastroth@med.uoa.gr (K.M.); mpikoul@med.uoa.gr (E.P.); pikoulianastasia@gmail.com (A.P.); evikakara@med.uoa.gr (E.K.); 2 Faculty of Humanities & Social Sciences, Open University of Cyprus, 2220 Nicosia, Cyprus; avra.sidiropoulou@ouc.ac.cy; 3 Department of Primary Education, University of Crete, 74100 Rethymno, Greece; avasiou@uoc.gr; 4 Department of Primary Education, University of the Aegean, 85132 Rhodes, Greece; mkladaki@aegean.gr; 5 Medical School, University of Nicosia, 2408 Nicosia, Cyprus; papantoniou.d1@live.unic.ac.cy

**Keywords:** well-being, creative health, resilience, participatory arts, crisis-affected, mixed-methods

## Abstract

**Background:** The COVID-19 pandemic disrupted school life worldwide, heightening risks to students’ psychosocial well-being and mental health, and creating an urgent need for sustainable support strategies during crises. Drama-based interventions, as participatory arts-based approaches, are proposed as flexible interventions that can strengthen resilience, social interaction, and emotional expression in school communities. **Objective**: This study evaluated the impact of a large-scale, short-term, remote drama-based intervention on the psychosocial adjustment and well-being of primary school students during the pandemic. **Methods**: An embedded mixed methods design with a pre-post measurement was employed, involving 239 teachers and 719 students aged 9–13 years from schools across various regions of Greece. Psychosocial functioning was assessed using a standardized instrument measuring levels of social, school, and emotional competence, as well as behavioral difficulties. The intervention, totaling 700 min over seven weeks, followed a five-day weekly structure that combined health-focused and psychosocial activities. **Results**: Quantitative findings indicated improvements across several dimensions of psychosocial adaptation and well-being, while Reliable Change Index analysis revealed important individual-level changes. Qualitative data corroborated these results, highlighting enhanced peer collaboration, increased emotional expression, and stronger classroom cohesion, while also emphasizing the adaptability and scalability of the approach under restrictive conditions. **Conclusions**: The findings suggest that such artful interventions can make a meaningful contribution to promoting well-being and sustaining the educational and social life of school communities during public health emergencies, thereby adding to the applied psychology evidence based on effective school health interventions.

## 1. Introduction

Large-scale crises, such as the COVID-19 pandemic, bring about significant disruptions in the daily lives of children and adolescents, affecting their social interactions, emotional balance, and school adjustment [1]. Prolonged restrictions, social isolation, and uncertainty about the future are factors that can heighten anxiety, undermine resilience, and lead to a decline in key socio-emotional functioning skills [2]. Within this context, supporting the mental health and psychosocial well-being of the student population constitutes a central priority in educational and social policy [3].

Schools, particularly during periods of emergency, serve not only as learning environments but also as stable points of reference for the provision of coordinated psychosocial support [4]. International guidelines for education in emergencies [5] and for psychosocial support and mental health [6] emphasize the importance of universally implementing emergency response programs designed to meet the needs of the school context, even when temporal and material resources are limited. Within this framework, a primary focus is placed on delivering meaningful support through targeted and pedagogically grounded interventions aimed at both the immediate alleviation of the psychosocial consequences of the crisis and the long-term strengthening of students’ resilience, socio-emotional competence, and integration within the school environment [1,7].

Drama-based interventions, as participatory arts approaches, have attracted growing research interest as potentially valuable interventions in this field [8,9,10,11]. Through experiential participation, collective creation, and the safe exploration of roles and scenarios, students process complex emotions, cultivate self-regulation skills, and develop essential communication and collaboration abilities that extend beyond the school setting [12,13,14]. Despite the expanding international literature [9], empirical evidence remains limited regarding the effectiveness of short-term, universal drama-based interventions (i.e., online or hybrid applications of drama pedagogy using digital platforms and interactive media), particularly those implemented in crisis contexts and through remote or hybrid formats, for enhancing resilience and social sustainability within school communities [15].

This study examines a short-term participatory, drama-based intervention implemented in schools during the period of social restrictions imposed by the COVID-19 pandemic. Employing a mixed-methods research design, it aims to capture changes in students’ psychosocial adjustment at both group and individual levels and to document teachers’ experiences, providing a comprehensive understanding of its effectiveness and implementation under crisis conditions. The present research is situated within the broader framework of educational and social sustainability, highlighting the role of applied art forms as strategic means of strengthening resilience within school communities.

### 1.1. Participatory Arts for Psychosocial Adjustment and Well-Being in Crisis-Affected Schools

During large-scale public health crises, such as the COVID-19 pandemic, the lives of children and adolescents are profoundly disrupted, with prolonged social isolation, interruption of school routines, and heightened uncertainty undermining their psychosocial functioning. These conditions negatively affect their social, emotional, and academic competence and increase the risk of behavioral problems [16,17,18,19,20]. Within this context, promoting social sustainability and collective resilience within school communities becomes a key prerequisite for sustainable development in education. Schools emerge as critical points of reference for the structured support of students’ mental health and development, emphasizing immediate, universal, and contextually adapted interventions designed to maintain functionality, prevent long-term negative impacts, and strengthen psychosocial adjustment [21]. In this study, psychosocial adjustment is defined as a child’s ability to respond effectively to the changing demands of social and school environments, maintain functional and supportive relationships with peers and adults, participate actively and consistently in the learning process, regulate emotions in ways that promote personal well-being, and demonstrate behaviors that facilitate cooperation and smooth integration into the group [22,23].

The international literature on Social and Emotional Learning (SEL) demonstrates that social inclusion, self-regulation, a sense of personal efficacy, and the development of meaning constitute core resilience mechanisms that enhance students’ adaptive capacity and support long-term improvements in behavior and psychosocial well-being through well-designed interventions [24]. During public health crises, such as the COVID-19 pandemic, these same factors act protectively, mitigating the impact of social isolation and emotional challenges while strengthening students’ ability to meet academic and social demands [25,26]. Within this framework, the drama-based approach represents a distinct and dynamic application of SEL principles through participatory art forms, as it integrates techniques that mobilize physical expression, imagination, empathy, and collective storytelling [27,28]. Interventions of this kind have been shown to strengthen skills of self-regulation, cooperation, and belonging, while fostering conflict resolution abilities and empowering student voice in group contexts [29,30]. Findings from the meta-analysis by Jiang and colleagues [9] confirm that programs utilizing theatrical activities, role-play, and collective creation through participatory arts significantly improve both socio-emotional skills and indicators of psychological resilience during public health crises. The outcomes are linked with the strengthening of social cohesion and sustainable communities, emphasizing the role of artistic practices in supporting the long-term stability of school ecosystems. Notably, the synthesis of this meta-analysis shows reductions in anxiety and depressive symptoms, as well as improvements in overall psychosocial well-being, with effects sustained into the post-crisis period [9].

Prior studies have shown that drama-based methods can facilitate the management of complex social issues, enhancing understanding and empathy even at very young ages [8]. Hatton [31] emphasized the contribution of drama to the cultivation of critical thinking and collective responsibility in relation to contemporary problems, while Turner-King & Smith [32] confirmed that applied theater provides a creative and locally adapted framework for processing the complexities of crises. During the COVID-19 pandemic, Tam [33] demonstrated that the integration of theatrical play, drama, and the arts significantly contributed to the restoration of psychological resilience upon the return to in-person education. Cziboly & Bethlenfalvy [12,34] and Gallagher et al. [35] highlighted the value of online drama-based sessions in enhancing communication, solidarity, and personal processing of the crisis under remote learning conditions.

Recent research has further contributed to the international evidence base, underscoring the importance of digital drama-based interventions for supporting bilingual literacy and psychosocial well-being in contexts of multiple crises [11], fostering resilience among children and adolescents during periods of crisis [36], and promoting psychosocial recovery following critical events [15]. The drama-based approach, by employing the concept of “esthetic distance,” creates a protective framework within which students can process difficult emotions without being overwhelmed psychologically [37]. Theatrical performance, within this approach, functions as a central mechanism for activating and mediating experiential learning, transforming artistic practice into a safe space for expression, experimentation, and meaning making [10]. In this way, experiential learning and meaningful engagement in activities are enhanced. During periods of crisis, interventions of this kind act as flexible practices capable of adapting to limited timeframes and available resources, contributing to the strengthening of psychosocial resilience in children and adolescents. From a sustainability perspective, such interventions provide both short-term relief and investment in the long-term resilience and social stability of school communities.

### 1.2. Assessing Short-Term School Interventions for Psychosocial Adjustment and Well-Being

In contrast to long-term school-based interventions, typically defined as those lasting six months or more and integrated into the school curriculum through multiple implementation phases [38], which are supported by extensive evidence and clearly defined evaluation criteria, short-term or emergency response interventions, particularly under crisis conditions, remain less studied and often lack universally accepted methodological evaluation standards [39]. This knowledge gap is especially critical during periods of widespread public health or social disruption, when schools are required to respond promptly to ensure the continuity of learning and the protection of students’ psychosocial well-being, while simultaneously contributing to the maintenance of social cohesion and community sustainability. These interventions often represent the only immediately available means of support before access to more systematic or long-term support structures can be established.

Even short-term or brief school-based social and emotional empowerment interventions can produce small to moderate effects (g ≈ 0.20–0.30) on key adaptation indicators, such as strengthening peer relationships, promoting prosocial behavior, and improving the sense of safety within the school, thereby contributing to the enhancement of school resilience in the face of crises [38]. In this meta-analysis [38], the authors reported improvements in peer relationships (g ≈ 0.23), prosocial behavior (g ≈ 0.19), and a stronger effect on school climate and perceived safety (g ≈ 0.30). At the level of overall school functioning, estimates indicate small but consistent effects (g ≈ 0.13), while academic performance shows effect sizes around g ≈ 0.11. The meta-analysis by Durlak and colleagues [40] confirms similar magnitudes for universal school interventions (academic performance g ≈ 0.27), highlighting that even short-duration programs can leave a measurable impact on academic outcomes. Regarding the emotional dimension, available data also indicate effects of g ≈ 0.12–0.24 in reducing emotional distress [40]. According to Hattie [41], an effect size in the range of 0.2–0.4 is roughly equivalent to one year of typical classroom instruction and represents the effect educators generally achieve through their everyday teaching practices.

Although the available data are encouraging, the evaluation of short-term interventions under crisis conditions still requires methodological strengthening. Beyond statistical significance and effect sizes, assessments should include indicators that reflect the practical or clinical significance of the observed changes, ensuring that findings have a tangible impact on school communities and support the maintenance of social sustainability. Within this framework, the Reliable Change Index (RCI) proposed by Jacobson and Truax [42] represents a widely recognized method, as it determines whether an individual’s performance change exceeds the range of fluctuations attributable to measurement error, considering the standard deviation, the reliability of the measurement instrument, and the individual’s initial score. Additionally, the literature suggests that a change equivalent to half or one standard deviation from the baseline measurement can be considered indicative of meaningful improvement [43], while a threshold of up to 1.5 SD is recommended for greater certainty [44]. The incorporation of confidence intervals in the interpretation of findings also provides an additional level of evaluation [45], as it reflects the magnitude of change and the precision of the estimate. This allows for an assessment of whether the observed outcome is likely to be replicable in the population and whether the recorded difference holds meaningful significance for educational practice.

### 1.3. Purpose and Research Questions

The present study aims to evaluate the effectiveness of an urgent remote drama-based intervention, grounded in participatory arts methodologies, for enhancing students’ mental health and psychosocial adjustment during prolonged social isolation imposed by the COVID-19 pandemic. The intervention was implemented across multiple schools in different regions of Greece and combined health and psychosocial activities with the goals of developing social skills, promoting emotional expression, strengthening school engagement, and fostering resilience under crisis conditions. This objective is particularly significant as it addresses two important gaps in international literature: the quantitative documentation of short-term school-based interventions during crises, and the systematic evaluation of drama and artistic approaches as structured tools for psychosocial support and the promotion of students’ psychosocial well-being and resilience. Based on the above, the study formulates the following research questions:

1. To what extent is students’ participation in the intervention associated with changes in social competence, emotional competence, academic competence, and the reduction in behavioral problems, considering both the magnitude and direction of change in each dimension?
2. To what extent do students demonstrate meaningful improvement in specific aspects of their psychosocial adjustment, with an emphasis on capturing change at the individual level?
3. Which specific areas of benefit emerge within each dimension of psychosocial adjustment, based on indicators reflecting enhanced social, emotional, academic, and behavioral skills, and how do these contribute to understanding the overall impact of the intervention?

## 2. Materials and Methods

### 2.1. Research Design

The study adopts an embedded mixed-methods design, integrating quantitative and qualitative data to provide a comprehensive evaluation of the intervention’s effectiveness [46]. The primary component of the study is quantitative, employing a one-group pre/post-test design to statistically capture and analyze changes across the various dimensions of psychosocial adjustment. The qualitative component is embedded complementarily through the analysis of teachers’ open-ended responses collected at post-test, to highlight areas of benefit and to interpret the observed changes.

The choice of a one-group pre/post-test design reflects the specific circumstances of the intervention context. The intervention was implemented during the COVID-19 pandemic, a period characterized by prolonged school closures and remote teaching, conditions that rendered random assignment or the creation of a control group ethically questionable and pedagogically inappropriate, as it would have required the deliberate withholding of potentially beneficial psychosocial support. As documented in the international literature [47,48,49], interventions during crises must adapt to contextual needs and ensure universal access, even if this limits the feasibility of implementing strict experimental protocols.

At the same time, the qualitative component, based on targeted open-ended questions administered at post-test, highlights the experiential and interpersonal mechanisms underlying the statistically observed changes. The integration of both types of data, within the study’s pragmatic philosophical framework, enhances both the interpretive power and the validity of the findings, ultimately providing a holistic understanding of the intervention’s impact under crisis conditions.

### 2.2. Participants

In the present study, 239 teachers from various educational regions of Greece participated. These teachers had taken part, during the COVID-19 pandemic and associated social distancing measures, in a remote training program offered through the Center of Continuing Education and Lifelong Learning (KEDIVIM) of the University of the Aegean. Within the context of the study, each teacher was asked to select three students from their class, based on sufficient familiarity with their behavior, in order to reliably complete the version of the scale designed for children aged 7 to 12, which relies on systematic observation. Teachers were instructed to select students who reflected a typical range of classroom behaviors and adjustment levels. Student selection was conducted by the teacher using random sampling, in accordance with the above criterion. To verify randomization and representativeness, basic demographic comparisons (gender, grade level, and teacher-reported academic standing) were performed, showing no significant deviations from the general classroom composition across participating schools.

In total, observational data were collected for 719 students, of whom 431 were boys (59.97%) and 288 were girls (40.03%). The mean age of the students was 10.82 years (SD = 0.96, Min = 9.18, Max = 13.72). Regarding grade level, 264 students (36.72%) were in fourth grade, 242 students (33.66%) in fifth grade, and 213 students (29.62%) in sixth grade. For the quantitative component of the study, sample adequacy was assessed using a power analysis with G*Power 3.1.9.7 [50]. The expected effect size for dependent samples (dz) was set at 0.20, based on meta-analyses of short-term school interventions in social-emotional learning and psychosocial empowerment, which report small to moderate effects (≈0.20–0.30) both prior to and during the COVID-19 pandemic [9,38,40,51,52,53,54]. The significance level was set at α = 0.05 and the desired power at 1-β = 0.95. Results of the analysis indicated that the minimum required sample size was 327 participants, demonstrating that the available sample of 719 students exceeds this requirement and ensures high statistical power for the planned analyses.

Of the 239 teachers who participated in the qualitative component of the study, the majority were women (82.85%), while male teachers accounted for 17.15%. Regarding employment status, 57.74% were permanent and 42.26% were substitute teachers, with teaching experience ranging from 8 to 28 years. Most teachers were primary education teachers (82.85%), with smaller proportions in parallel support teachers (10.46%) and special education teachers (6.69%). In terms of teaching assignment, 25.94% worked in fourth grade, 23.85% in fifth grade, 27.62% in sixth grade, 15.90% in special inclusion classes, and 6.69% in educational priority zone classes. Regarding academic qualifications, 24.27% held only their basic degree, 16.32% had received additional training exceeding 200 h, 5.44% held a second degree, 52.72% had a master’s degree in an education-related field, and 1.26% held a doctoral degree.

### 2.3. Instruments

For the collection of quantitative data, the standardized Psychosocial Adjustment Test [55] was used. This instrument is designed to assess the psychosocial functioning of children in preschool and school age. In the present study, the teacher-report version for students aged 7 to 12 years was applied. The tool is completed by teachers and includes 112 items, rated on a five-point Likert scale, assessing the frequency of specific behaviors related to children’s adjustment in school and social environments.

The structure of the test is organized into four main subscales: social competence (27 items), academic competence (29 items), emotional competence (25 items), and behavioral problems (31 items).

*Social competence* is assessed through three interrelated dimensions. Assertiveness and leadership ability (6 items) examines the extent to which children take initiative and assume responsibility roles in the classroom (e.g., “Expresses their opinion when needed to find a solution”). Interpersonal communication (15 items) focuses on behaviors and skills that lead to positive social outcomes (e.g., “Responds when asked something”). Peer cooperation (6 items) refers to active participation in joint activities and achieving collective goals (e.g., “Invites other children to participate in activities”).

*Academic competence* covers four dimensions. Motivation (4 items) assesses the student’s desire to achieve learning goals (e.g., “When starting something, insists on completing it”). Organization and planning (13 items) relate to behaviors that help the student plan and complete tasks within deadlines (e.g., “Completes assigned tasks”). Academic effectiveness (6 items) includes behaviors that contribute to overall student performance (e.g., “Is a good student”). School adjustment (6 items) refers to smooth integration and functioning in the school environment (e.g., “Participates in class activities”).

*Emotional competence* is evaluated across four parameters. Self-control (8 items) refers to regulation of emotional reactions (e.g., “Controls their anger”). Emotion management (5 items) assesses the child’s ability to recognize and regulate their emotions (e.g., “Shows others how they feel”). Empathy (5 items) involves understanding the emotions of others (e.g., “Usually understands how others feel”). Stress management (7 items) relates to the use of self-regulation strategies in stressful situations (e.g., “Remains calm when problems arise”).

*Behavioral Problems* subscale includes three dimensions. Interpersonal adjustment (9 items) concerns the exhibition of aggressive or reactive behaviors (e.g., “Reacts strongly during arguments”). Intrapersonal adjustment (9 items) relates to the presence of emotional difficulties (e.g., “Is afraid of making mistakes”). Hyperactivity, impulsivity, and attention difficulties (12 items) focuses on behaviors involving impulsive reactions or lack of concentration (e.g., “Overreacts to minor stimuli, is an impulsive child”). A distinctive feature of this subscale is that, unlike the others, higher scores correspond to lower levels of adjustment.

The instrument has psychometric validation for the age group under study, having been developed and standardized on a Greek student population aged 10–12 years, covering a wide range of socioeconomic and geographic contexts. Internal consistency indices (Cronbach’s α) range from 0.72 to 0.95 for the four main subscales, while values for the individual dimensions remain at acceptable levels. Test–retest reliability demonstrates stability of the results over time. Construct validity has been supported through factor analysis, which confirms the four-factor structure, as well as through comparisons with related assessment tools for child adjustment. The standardized nature of the test allows for conclusions at both group and individual levels, facilitating the analysis of change before and after the intervention.

For the collection of qualitative data, a set of four open-ended questions was employed, each corresponding to one of the main dimensions of psychosocial adjustment: social competence (“What changes have you observed in your students’ cooperation and interaction with their peers?”), academic competence (“What changes have you noticed in the organization of schoolwork and overall response to learning demands?”), emotional competence (“What changes have you observed in students’ management of their emotions?”), and behavioral problems (“Have you observed changes in behaviors that hinder certain students’ adjustment in the digital classroom?”). The questions were phrased in a neutral and broad manner, allowing teachers to describe specific incidents, observations, or changes they identified in their students during and after the intervention. This approach aimed to capture teachers’ experiences and perceptions of the intervention’s impact, thereby creating a complementary qualitative component directly linked to the dimensions measured in the quantitative part of the study.

### 2.4. Data Collection

The data collection process was conducted in discrete stages to ensure scientific validity and ethical compliance. Initially, the necessary approval was obtained from the Department of Primary Education at the University of the Aegean (Approval No. 619-2021), covering both the implementation of the research and the application of the intervention within the school environment. Subsequently, prior to the start of the intervention, participating teachers attended a short, structured training program at the KEDIVIM of the University of the Aegean, where they were trained in the proper and standardized completion of the Psychosocial Adjustment Test [55] (see Section 2.3), ensuring accuracy and consistency in data recording.

Immediately after the training, each teacher randomly selected three students from their class with whom they were sufficiently familiar and completed the observation tool for these students, recording their initial psychosocial profile. To protect personal data, each student was assigned a unique alphanumeric code, which was used throughout all stages of data collection and analysis. Subsequently, the teachers, under the guidance and continuous support of the interdisciplinary research team, implemented the planned remote intervention, adhering to the established fidelity principles. The intervention was delivered within a uniform framework and schedule across all participating schools, while the research team ensured consistency and quality of implementation.

Upon completion of the intervention, the teachers re-administered the same instrument for the same students to quantitatively capture changes in psychosocial adjustment levels. This process provided teachers with data on these cases, grounded in scientific principles of change measurement, which they could use to reflect upon and evaluate the effectiveness of their own instructional intervention. The data, organized by anonymized code and accompanied by basic demographic information of the students, were submitted to the research team for further analysis. The team calculated the initial cumulative scores for all responses received for each dimension and converted them into standard scores according to the instrument’s scoring guidelines.

Simultaneously, at this stage, the teachers responded to four open-ended questions corresponding to the main dimensions of the instrument (social competence, academic competence, emotional competence, behavioral problems; see Section 2.3), describing specific incidents and changes they observed in the students during and after the intervention. These responses formed the basis of the qualitative component of the study, complementing the quantitative data and providing the researchers with the opportunity for interpretive deepening of the findings.

### 2.5. Intervention

The intervention was designed based on the principles of Social and Emotional Learning (SEL) and the promotion of psychosocial resilience, utilizing the dynamics of drama-based pedagogy and participatory forms of art, in accordance with recognized international standards for intervention design in emergency contexts, such as the IASC Guidelines on Mental Health and Psychosocial Support in Emergency Settings [6] and the Minimum Standards for Education: Preparedness, Response, Recovery [5].

The core of the design included the drama-based method of reader’s theater, implemented through a technologically mediated instructional format, inspired by classical stories and fairy tales, as well as the use of dramatic and theatrical techniques with psychosocial and/or health-related objectives. The activities aimed to enhance students’ psychosocial functioning through the development of social skills, the cultivation of emotional expression and empathy, the promotion of teamwork, and the understanding of collective storytelling, while simultaneously fostering self-regulation and metacognitive skills. Within this framework, health-related activities were defined as those promoting mental health and emotional well-being, such as exercises in emotion recognition and management, emotional release activities, and relaxation techniques. Psychosocial activities, on the other hand, were considered those that strengthened social relationships, the sense of belonging, and cooperation, including drama games, improvisational theater, and group dramatizations of scenes, which promoted collective creation and interaction among students (see Table 1).

The intervention was implemented as a short-term, intensive program, organized into a five-day cycle of digital sessions lasting 20–30 min each day via the Webex platform. This cycle was repeated weekly over a period of seven weeks. Each participating class functioned as a single intervention group of approximately 15–25 students, and all students in the class took part in the activities, even though quantitative assessments were conducted only for three randomly selected students per class. The program combined health-promoting activities, broadly aimed at fostering students’ mental well-being, with psychosocial activities designed to enhance group cohesion and social inclusion. Several activities served a dual purpose, simultaneously supporting psychological well-being and collaborative learning, in line with internationally recognized standards for the design of school-based interventions in crisis contexts.

### 2.6. Intervention Fidelity Assessment

The implementation was assigned to teachers who, during the specified period, participated in a structured training program conducted within the framework of the KEDIVIM at the University of the Aegean. The training included theoretical instruction on the principles and objectives of the intervention, detailed guidance on the content and structure of each session, practical exercises in the activities, as well as training in the use of the tools within the remote learning environment. To ensure intervention fidelity in the digital environment, a structured implementation framework was followed. Teachers responsible for delivering the intervention attended a training workshop, where they received detailed instructions and guidance on the data-based methods to be employed, as well as directives for integrating these methods within digital teaching conditions. In parallel, they were provided with guidelines and sample lesson plans to facilitate preparation and consistent implementation of the intervention.

During the implementation period, weekly online feedback meetings were conducted with the interdisciplinary research team, during which the progress of the intervention was documented, emerging issues were discussed, and alignment with the original plan was verified. Additionally, teachers had access to an online communication network, enabling them to exchange experiences, discuss observations, and share practices, thereby reinforcing collective support and fostering a sense of community. Fidelity assessment involved the systematic documentation by teachers of the activities implemented, their duration, and any deviations from the planned schedule, using a weekly checklist. This checklist, designed in alignment with the implementation framework, recorded both adherence to the protocol and the quality of activities. Additionally, the research team conducted a classroom observation during the intervention period to verify methodological compliance. These observations were documented using a standardized checklist, allowing for the evaluation of the degree of adherence to the intervention plan. Fidelity documentation and observation feedback confirmed that the intervention was implemented with high adherence and consistency across all participating classrooms. Based on the weekly fidelity checklists, adherence to the planned structure exceeded 90% for most sessions, with only minor deviations primarily related to time management or digital connectivity constraints.

### 2.7. Data Analysis and Intervention Evaluation

For the implementation of the present research phase, in addition to the prior approval granted by the University of the Aegean, a specific authorization was obtained from the Research Ethics and Deontology Committee of the Medical School of the National and Kapodistrian University of Athens (No. 242/2025).

For the quantitative data, an initial preprocessing phase was conducted. Minimal missing values (<1% per variable) were imputed using group mean substitution, while outliers were assessed through a combination of boxplots and standardized residuals, with no cases requiring exclusion. Data distribution was visually inspected using Q-Q plots, leading to the application of parametric tests, which were performed using Jamovi (v. 2.6.44). The evaluation of the intervention at the group level was conducted using paired-samples *t*-tests to compare the two measurement phases (pre-test vs. post-test) for each dimension of psychosocial adjustment. For each dimension, mean differences, 95% confidence intervals, and effect sizes (Cohen’s d) were calculated to assess the practical significance of the findings. Consequently, within the context of short-term school interventions under conditions of social restrictions, d values of approximately 0.20–0.30 for social competence, academic competence, emotional competence, and reduction in behavioral problems are interpreted as educationally meaningful and realistically expected, in line with the relevant literature [38,54,56,57,58,59].

For the assessment at the individual level, the RCI (Jacobson & Truax, 1991) [42] was calculated for each student and for each dimension using R (v. 4.5.1). This index expresses whether the observed change in a student’s score exceeds what could be expected by chance or measurement error. RCI values greater than +1.96 indicate statistically reliable improvement, whereas values below −1.96 indicate reliable deterioration. Values within the range [−1.96, +1.96] suggest that the change cannot be considered reliable [60]. Additionally, the proportion of participants with initially high scores (ceiling effect) was recorded, as this may limit the scope for measurable improvement [61]. High baseline scores were defined as those at or above the 90th percentile of the pretest distribution for each subscale too [61]. To estimate the true potential for improvement, an Adjusted Improvement Index was also calculated, which corrects the observed improvement percentage by taking the ceiling effect into account, according to the following formula:

(1) $$\mathrm{Adjusted}\;\mathrm{Improvement}\;\left(\%\right)=\frac{\mathrm{Improvement}\;\left(\%\right)}{100-\mathrm{Ceiling}\;\mathrm{Effect}\;\left(\%\right)}\times 100$$

This approach allows for a more precise assessment of intervention effectiveness, particularly when a substantial proportion of participants begin with high baseline scores. The results were visualized using boxplots to depict pre-post changes for each dimension, and scatter plots to illustrate the relationship between pre- and post-assessment scores as well as the change category according to the RCI.

In the qualitative component, the analysis followed the framework of Interpretative Phenomenological Analysis, applying a theory-driven thematic analysis, with the conceptual model of child and adolescent psychosocial adjustment developed by Chatzichristou et al. [55] serving as the interpretative lens. The procedure involved repeated readings of the teachers’ written responses to identify meaningful units, recurring patterns, and interpretative connections. In the initial stage (open coding), all emerging concepts were recorded without restriction. This was followed by axial coding, in which the initial codes were grouped into subcategories based on thematic and conceptual similarity. In the final stage, selective coding contributed to the synthesis and organization of subcategories into broader thematic axes, fully aligned with the primary dimensions and subdimensions of the psychosocial adjustment conceptual model underpinning the employed instrument. In cases of conceptual overlap, individual codes were merged for analytical coherence. The entire process was conducted using the qualitative analysis software QCAmap (version 1.2.0). The analysis resulted in the identification of ten initial codes, which, through grouping and conceptual integration, were consolidated into four broader sub-axes reflecting the main dimensions of psychosocial adjustment.

## 3. Results

### 3.1. Changes in Core Dimensions of Psychosocial Adjustment

**Figure 1:**
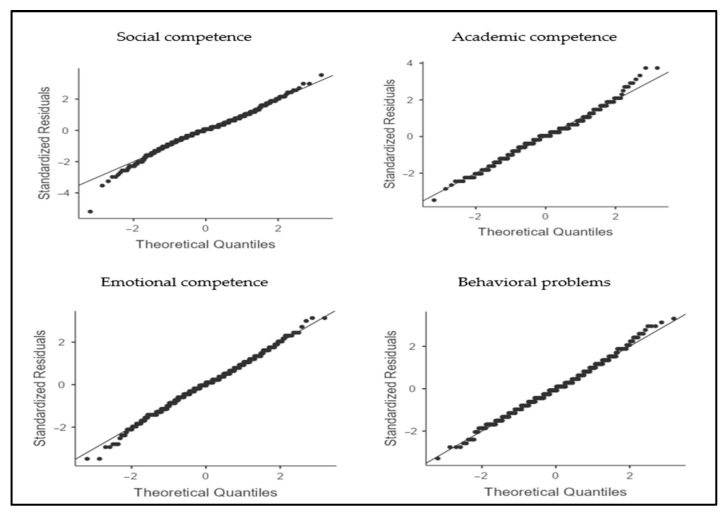
Q-Q plots of residuals for each dimension of psychosocial adjustment.

Prior to conducting the paired-samples *t*-tests, the assumptions for their application were examined. Visual inspection of the Q-Q plots indicated that the differences between measurements adequately approximated normality, with no extreme values being detected (Figure 1). Therefore, the use of parametric analyses was deemed appropriate for evaluating changes across the four dimensions of psychosocial adjustment.

Based on the descriptive statistics, students appeared to show improvement in their social, academic, and emotional competence, while, at the same time, behavioral problems decreased (see Table 2).

Specifically, social competence increased from M = 51.02 (SD = 10.21) to M = 52.54 (SD = 9.64), academic competence from M = 46.22 (SD = 8.26) to M = 47.36 (SD = 7.53), and emotional competence from M = 48.71 (SD = 10.31) to M = 50.43 (SD = 10.01). In contrast, behavioral problems decreased from M = 53.16 (SD = 8.24) to M = 50.70 (SD = 9.41). The results of the paired-sample *t*-tests indicated that the observed changes were statistically significant across all dimensions of the psychosocial adjustment model, confirming the effectiveness of the drama-based intervention (see Table 3).

**Figure 2:**
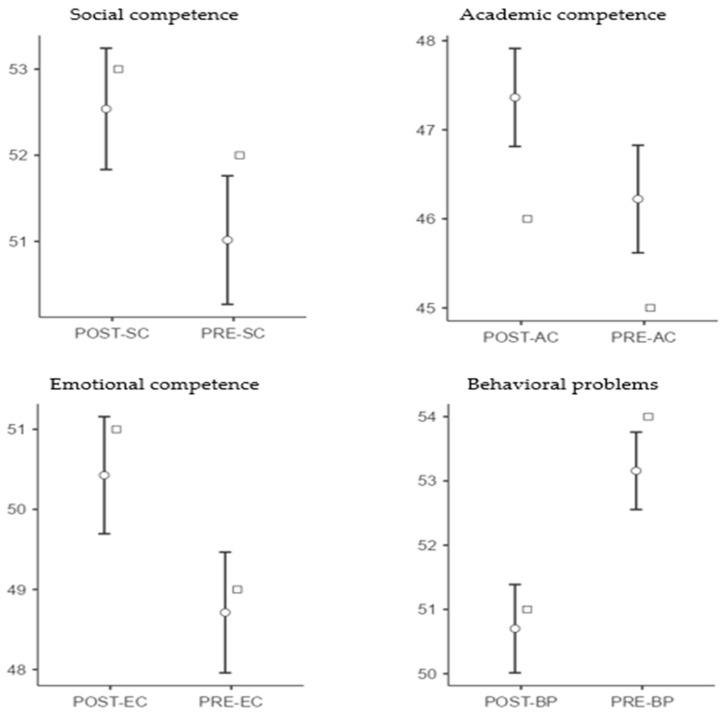
Graphical representation of pre-test and post-test measurements with 95% confidence intervals for the dimensions of psychosocial adjustment. ***Note***. ○ = Mean (95% CI); □ = Median.

Specifically, social competence showed a statistically significant increase (*t*(718) = 5.66, *p* < 0.001, *M* difference = 1.52, 95% CI [0.99, 2.05], *d* = 0.21). Academic competence also improved significantly (*t*(718) = 6.30, *p* < 0.001, *M* difference = 1.14, 95% CI [0.79, 1.50], *d* = 0.24). Emotional competence likewise demonstrated an increase (*t*(718) = 6.36, *p* < 0.001, *M* difference = 1.71, 95% CI [1.19, 2.24], *d* = 0.24). Regarding manifestations of behavioral problems, a statistically significant decrease was observed (*t*(718) = −11.74, *p* < 0.001, *M* difference = −2.46, 95% CI [−2.87, −2.05], *d* = −0.44) (see Figure 2).

### 3.2. Reliable Individual Variation and Student Distribution

**Figure 3:**
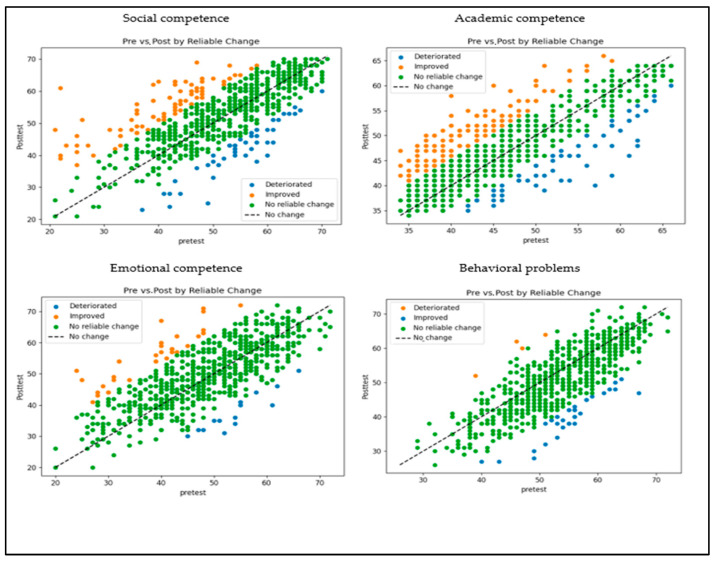
Pretest-posttest relationship for each student and classification of change according to the Reliable Change Index across dimensions. *Note*. Green indicates the absence of reliable change (stability), orange represents reliable improvement, and blue denotes reliable deterioration. The diagonal line represents the point of zero change.

To evaluate whether the changes observed between pretest and posttest reflect genuine individual change rather than measurement error, the Reliable Change Index (RCI) was computed for each student, following the methodology proposed by Jacobson and Truax [42]. Figure 3 illustrates the pretest–posttest relationship for each student, with a color-coded scheme representing levels of reliable improvement, deterioration, or non-reliable change.

The majority of students demonstrated stable performance (82.06% for social competence, 74.41% for academic competence, 93.05% for emotional competence, and 94.71% for behavioral problems). Improvement, defined as an increase greater than +RCI, or a decrease in the case of behavioral problems, was observed in 11.54%, 17.11%, 5.01%, and 4.73% of students, respectively. In contrast, deterioration (<−RCI) was observed in 6.40% of students for social competence, 8.48% for academic competence, 1.95% for emotional competence, and 0.56% for behavioral problems.

Given that high initial scores can constrain the potential for improvement (ceiling effect), the proportion of students scoring ≥ 90th percentile at pretest was examined. This proportion was 11.54% for social competence, 12.66% for academic competence, 11.13% for emotional competence, and 10.88% for behavioral problems. Table 4 presents a summary of the RCI values, as well as the percentages of improvement, stability, deterioration, and ceiling effect for each dimension.

To mitigate the impact of the ceiling effect, the percentage of improvement was calculated only for students with available room for improvement (Adjusted Improvement). RCI values ranged from 5.31% for behavioral problems to 19.59% for academic competence, with social competence at 13.04% and emotional competence at 5.64%.

### 3.3. Qualitatively Documented Areas of Benefit Across Dimensions

The analysis of the qualitative data led to the grouping of findings into thematic dimensions corresponding to the main domains of psychosocial adjustment. Each dimension includes codes, capturing more specific aspects of the students’ experiences, as reported by the teachers. The following table summarizes the dimensions, codes, and their content (see Table 5).

At the level of social competence, teachers observed increased willingness to participate and collaborate, particularly through group drama-based activities and the joint preparation of the performance. Children who had previously avoided group interaction appeared to find a role and voice through collective storytelling. As one participant noted: “*During the rehearsal of the choral dialogue, even the most reserved students participated actively to ensure the scene was performed correctly*” (Participant 84). Regarding social assertiveness, role-assignment activities enhanced students’ confidence and ability to express requests respectfully: “*During the role distribution, a student who was usually silent eagerly requested to play the narrator, as they wanted to try something new*” (Participant 137).

With respect to academic competence, the demands for organization, consistency, and creative engagement involved in preparing the performance appeared to be transferred to other learning activities. “*Students who often forgot their assignments remembered to bring their props without any reminder*” (Participant 29). Additionally, the creative development of texts and roles increased interest in the subject: “*They read the text at home not because I asked them to, but because they wanted to propose ideas for changes to the lines and to present it better*” (Participant 212).

Regarding emotional competence, the drama-based process served as a safe context for recognizing, expressing, and regulating emotions. Improvisations in high-tension scenes helped students experiment with alternative conflict-resolution strategies. As one teacher described: “*During the improvisation of the conflict scene… a child who usually reacted intensely suggested resolving the issue through dialogue, because ‘we don’t need to fight on stage to show the problem*” (Participant 56). Emotion regulation was also reinforced through relaxation activities prior to the performance: “*Before going on the digital ‘stage,’ all the children practiced the deep-breathing exercises we had learned together and entered the flow more calmly*” (Participant 198).

Finally, concerning the behavioral problems, teachers observed that dramatic and theatrical techniques led to more disciplined participation and moderated impulsive reactions, particularly during theatrical games requiring collaboration and respect for others’ time. “*In the ‘frozen image’ game, a student who usually jumped in waited silently to be assigned a role, so as not to disrupt the group composition*” (Participant 102). Furthermore, collaborative creation scenes reduced conflicts: “*Two students who often argued decided on their own during rehearsal to play siblings together…*” (Participant 176).

## 4. Discussion

The aim of the present study was to examine the extent to which a short-term school-based intervention, designed to support mental health and psychosocial well-being through participatory art forms with a central focus on drama-based approaches, can enhance key aspects of students’ psychosocial adjustment during the period of social isolation imposed by the COVID-19 pandemic. The evaluation combined quantitative indicators to capture changes at the group level, measures to identify meaningful individual-level changes, and qualitative data from the teachers who implemented the intervention, in order to provide a comprehensive assessment of the intervention’s effectiveness within this unique public health context. The findings indicated improvements in students’ psychosocial adjustment by offering targeted support during a period of heightened uncertainty and limited social interaction. Qualitative insights complemented the statistical analysis, deepening the understanding of how the intervention influenced students’ adjustment and well-being.

Beyond confirming patterns already reported in arts-based and SEL research, the present study provides new evidence on how participatory drama can function as a structured psychosocial mechanism during a large-scale public health emergency. By addressing social, emotional, academic, and behavioral domains simultaneously, the findings expand current knowledge on the multidimensional effects of school-based arts interventions and clarify how these mechanisms operate both at group and individual levels.

### 4.1. Effectiveness for Social Competence and Peer Relationships During Crisis-Related Isolation

The present study demonstrated that students’ participation in the emergency-response drama-based intervention was associated with improvements in their social competence, reflecting the range of effects documented internationally for short-term social-emotional learning interventions during crisis periods. This improvement aligns with the findings of a recent meta-analysis [38], which reported similar positive changes in peer relationships and prosocial behavior in universal school-based applications. Particularly under conditions of social isolation, such as during the COVID-19 pandemic, these seemingly modest improvements in social functioning indicators acquire heightened educational and psychosocial significance, as they suggest the restoration of relationships, enhanced collaboration, and the reactivation of peer connections [26]. Although substantial individual changes did not concern the entire participant group, for a subset of students, the intervention experience functioned positively, providing a context for re-engagement and the development of social skills that had been eroded due to the prolonged social isolation imposed by the pandemic.

The interpretation of the quantitative findings is reinforced by the qualitative analysis, which highlighted two main areas of improvement: the development of a collaborative stance and the enhancement of social assertiveness. In the domain of collaborative stance, teachers observed that participation in group drama-based activities fostered a safe environment in which students shared responsibilities, undertook joint tasks, and developed a sense of common purpose. This condition, which promotes mutual support and synchronization, has also been described in other studies of participatory art forms during the pandemic as crucial for reconnecting students with the social structures of the school community [9,11,13,35].

Concurrently, social assertiveness was enhanced through activities that allowed students to express desires and assume roles in ways that maintained respect for others. These activities provided a protected “esthetic distance” framework within which students could experiment with new forms of social behavior without fear of social consequences, consistent with findings from drama therapy and educational theater interventions implemented in similar contexts [15]. International literature recognizes that such interventions, even when brief, can act as social accelerators, enhancing trust, empathy, and a sense of belonging [9]. This particular intervention, with its phased structure and clear focus on collective creation, appears to leverage these mechanisms in a way that enables the transfer of skills developed within theatrical activities to everyday remote school interactions. Beyond improving immediate social functioning, this outcome contributes to the social sustainability of the school community by strengthening the maintenance of cohesive and supportive relationships, which are foundational for resilience and collective recovery during periods of extended crisis. This study further advances current knowledge by showing how collaborative theatrical processes can preserve peer connection and social reciprocity even within remote learning environments, providing insight into the psychosocial mechanisms that sustain social bonds during health emergencies.

### 4.2. Effectiveness for Academic Competence and School Functioning During Crisis Disruption

The observed improvement in academic competence and school functioning, although not encompassing all students, falls within the range of effects documented internationally for short-term social-emotional learning interventions in school settings, particularly during public health crises. According to the literature, interventions of this type consistently achieve small but meaningful gains in school functioning and academic performance, with evidence of greater impact when school climate and a sense of safety are reinforced [38,59]. The present intervention appears to align with this pattern, as a proportion of students demonstrated substantial progress, while the majority maintained stable levels of functioning despite the disruption caused by the pandemic.

This improvement was observed not only at the general level of academic competence but also in more specific aspects of school functioning, as highlighted through teachers’ qualitative reports [20]. In particular, adaptability to schoolwork (academic engagement) appeared to be markedly enhanced, with students demonstrating greater consistency in task completion, more active participation, and improved time management. This finding can be linked to the way the drama-based approach cultivates skills in discipline, collaboration, and adherence to a shared plan through creative processes, skills that transfer functionally to the school context. Consistent with previous research, engagement in artistic, collaborative activities creates an environment in which active participation is tied to the success of the final outcome, thereby enhancing school engagement and competence even under remote learning conditions [11,62].

Similarly, learning motivation was enhanced through the experience of assuming roles and contributing creatively to activities that required preparation and responsibility. The drama-based methodology, utilizing dramatized storytelling, theatrical improvisation, and embodied expression, provided learning conditions that simultaneously engage cognitive, emotional, and social domains, thereby fostering students’ interest and intrinsic commitment. Consistent with international research on the implementation of artistic interventions during periods of crisis, this engagement appears to function as a stabilizing mechanism, particularly in environments where the sense of routine and purpose has been disrupted [31,32,36]. Beyond immediate learning improvements, the enhancement of academic competence and school functioning is linked to long-term educational and social sustainability, as it supports the maintenance of stable learning processes and the reconstruction of the school community during periods of prolonged crisis. This aspect suggests that drama-education can serve as a promising pedagogical approach for supporting the resilience of the school system. What distinguishes this intervention is its demonstration that drama-based engagement can maintain students’ learning regulation and intrinsic motivation amid large-scale public health disruption, pointing to its value as a feasible and low-intensity means of supporting educational continuity and student well-being.

### 4.3. Effectiveness in Terms of Emotional Competence During Periods of Crisis

The present intervention demonstrated improvements in emotional competence, with an effect size falling at the upper range of small to moderate effects (≈0.12–0.32) reported internationally for short-term school-based social-emotional learning interventions during periods of crisis and public health restrictions. The international literature indicates that even moderately scaled programs can substantially contribute to reducing emotional distress and enhancing emotion-regulation skills. The findings of the present study align with these observations, confirming that the drama-based approach can operate effectively even within a brief timeframe and under remote learning conditions.

The analysis of qualitative data provided deeper insight into the mechanisms likely underlying these improvements. Self-regulation was enhanced as students were required to adjust their behavior and expression to meet the demands of a role or drama-based activity. The continuous alternation between preparation, performance, and feedback created a repeated pattern of impulse control, energy regulation, and group adaptation. Similar results have been reported in studies within education and SEL programs, where participatory a er clarifies the active mechanisms of drama-based intervention forms provide safe environments for practicing self-regulation skills that transfer beyond the creative process [37,63,64,65,66].

Empathy appears to have been strengthened through activities requiring the adoption of different perspectives and the interpretation of characters’ emotions. This process, central to acting and storytelling, fosters the ability to recognize and interpret both verbal and non-verbal cues of others, as confirmed by previous educational theater interventions [67]. During the pandemic, when physical contact and social interaction were restricted, this experiential practice served as a substitute for social experience, mitigating the negative consequences of isolation on the development of empathy.

Emotion management improved as students were exposed to diverse emotional situations within a controlled and supportive environment. The weekly progression of the interventions, from preparation and improvisation to performance, provided opportunities to recognize, label, and regulate emotions. SEL literature emphasizes that conscious emotion recognition and regulation constitute fundamental skills for psychological resilience during crises, and the present findings identify them as one of the core components of the intervention [68,69,70,71,72,73]. A novel contribution of this study lies in illustrating how embodied drama activities can serve as emotional regulation tools in remote learning contexts, offering evidence that theatrical embodiment may operate as a protective factor for emotional balance during crisis-related isolation.

Stress coping was particularly exercised in activities incorporating movement, bodily expression, and humor. The theatrical act functioned as a release mechanism, while collaborative creation enhanced the sense of support from the group. Consistent with research on psychological support during crises, providing a framework that simultaneously allows expression, and social cohesion facilitates effective stress management, reducing symptoms of emotional tension [74,75,76,77].

### 4.4. Effectiveness in Reducing Behavioral Problems

The intervention recorded a reduction in behavioral problems at the group level, with an effect size that, although moderate, corresponds to outcomes typically associated in the international literature with longer-term programs [41]. The fact that the present intervention was short-term makes this finding particularly noteworthy, suggesting that drama-based practices, when well-structured and focused, can foster noticeable improvements in classroom behavior even within a brief period. Although only a small proportion of students demonstrated reliable individual improvement, the positive shift across the entire group, as indicated by the effect size, demonstrates that the intervention benefited overall classroom climate and behavior management in the online learning environment. This finding aligns with research showing that, even when significant individual changes are limited, overall reductions in problematic behaviors at the group level can have an immediate pedagogical and psychosocial impact [78].

The qualitative analysis identified key areas of improvement as reductions in hyperactivity/impulsivity and decreases in aggressive or intrusive behavior. The reduction in hyperactivity can be attributed to the structure of the theatrical activities, which combined controlled physical expression with a clear framework of rules and goals. Within this framework, physical energy was channeled into creative processes requiring alternation between activation and calm phases. International literature on arts-based and creative health interventions for students with high levels of motor restlessness has shown that the combination of physical activity and structured guidance can reduce impulsivity, improving self-control [79].

The reduction in aggressive or intrusive behavior can be explained by the empathy and social interaction mechanisms [80], activated in drama-based exercises. Role alternation, adopting the perspectives of other characters, and collaboratively building scenes foster mutual respect and recognition of others’ boundaries. Other research has linked the enhancement of prosocial behavior through creative, collaborative activities to reductions in aggressive responses, particularly among student groups experiencing heightened stress or disruption due to external factors, such as public health restrictions [80,81]. The systematic reduction in behavioral problems, even in short-term interventions, can be considered foundational for the creation of a stable and supportive learning environment. Strengthening a positive school climate contributes both to immediate educational functioning and to the long-term sustainability and resilience of school communities, particularly during periods of heightened social or public health uncertainty. Unlike most previous short-term interventions, this program shows that structured participatory arts can yield measurable behavioral improvements even in emergency education conditions, thereby expanding the evidence base for arts-based strategies in school mental health promotion.

## 5. Limitations and Future Research

The present study, although employing a mixed-methods approach that enhanced the validity of its conclusions, carries certain limitations that should be considered. First, the use of a single sample without a control group limits the ability to infer causal relationships between the intervention and the observed changes, although this choice was justified. Specifically, the absence of a control group restricts causal inference, as the observed improvements cannot be fully separated from potential external influences (e.g., general school adaptation, recovery trends following the pandemic period, etc.). However, the combination of quantitative and qualitative data provides convergent evidence supporting the internal coherence and plausibility of the reported effects. Second, data collection relied on self-reports and teacher assessments, which may be influenced by socially desirable responses [82]. Third, the implementation of the intervention under pandemic and crisis conditions, with restrictions on physical presence and variations in participation intensity, may have affected both the delivery and effectiveness of the activities, despite measures taken to ensure fidelity of implementation.

For future research, it is recommended to employ experimental or quasi-experimental designs with control groups to strengthen internal validity. Expanding the sample size and implementing the intervention across diverse sociocultural contexts could enhance the generalizability of the findings. Particularly important is the comparative evaluation of short-term and long-term immediate-response programs during crisis conditions, especially those based on applied art forms such as drama-based education and other participatory creative practices. Such an approach would allow for the investigation of both immediate and long-term effects across cognitive, emotional, and social domains.

Simultaneously, the development and standardization of effectiveness criteria for emergency-response interventions targeting children and adolescents is deemed necessary. Integrating these criteria into an internationally recognized evaluation framework could represent a strategic step toward establishing a repository of evidence-based practices that can be deployed immediately in future crises. The creation of such protocols would facilitate both the comparability and reproducibility of interventions and support the sustainability and resilience of school communities, ensuring continued access to high-quality psychosocial support even under uncertain conditions.

## 6. Conclusions

The present study highlighted that the drama-based approach, when incorporated into short-term school interventions, may positively contribute to students’ mental health, psychosocial adjustment, and social cohesion, particularly for those experiencing prolonged social isolation. Its effectiveness lies in combining structured health and psychosocial activities with an artistic and participatory perspective that allows for a high degree of creative freedom, thereby creating a safe and supportive environment in which students can process experiences, express emotions, and regain a sense of connection with the community. This reconnection extends beyond the individual, functioning as a mechanism for strengthening the overall resilience of the school community and supporting social sustainability during periods of crisis. Under pandemic conditions, as well as in other forms of crises that disrupt social and educational functioning, this methodology provides both pedagogical and psychosocial benefits, enhancing resilience and the capacity for collective recovery [15], while laying the groundwork for the long-term reinforcement of bonds and collaboration within the school system.

Based on the findings, it can be posited that applied theater in education and emergency response, as a form of participatory art, can bridge the gap between formal learning processes and creative health support frameworks, functioning as an integral component of a holistic intervention strategy in emergency contexts [10]. By enhancing skills such as self-regulation, empathy, emotion management, and collaboration, students acquire resources that extend beyond academic success to include the capacity to manage the psychological impacts of crises and contribute to collective recovery and community resilience. The role of theatrical performance is highlighted as a mediating process, capable of bridging experiences, needs, and resources across multiple levels, a feature that can form the foundation of a coherent theoretical framework, provisionally described as the “Mediated Theatrical Performance Theory”.

This dual contribution of theatrical performance, impacting both educational and psychosocial domains, renders the method relevant for integration into formal crisis management policies and practices. The approach aligns fully with international standards for emergency interventions, such as the IASC Guidelines on Mental Health and Psychosocial Support in Emergency Settings [6] and the INEE Minimum Standards for Education: Preparedness, Response, Recovery [5], which emphasizes the creation of safe, coherent, and psychologically supportive learning environments. In practice, drama-based methods can be embedded within national and local social sustainability strategies, educational emergency protocols, public health plans, and professional development programs for teachers and psychosocial support personnel, ensuring that schools remain points of reference and solidarity when communities are under strain.

## Figures and Tables

**Table 1:** Weekly Structure of Health-Related and Psychosocial Activities in the Emergency Educational Response Intervention.

Day	Goal/Gradual Release Stage	Health-Related Activities	Psychosocial Activities	Connection to Final Performance
Monday	Modeling—Teacher demonstrates and guides	“Emotional Pulse of the Group”— recognition and expression of emotions	Opening theater game—engagement and team building	Introduction to the classic story forming the week’s basis
Tuesday	Guided Practice- Students participate with support	“Hot Seating”— development of empathy and emotion regulation	Group monologs based on story themes	Deepening understanding of characters and plot
Wednesday	Gradual Transfer of Responsibility- Students take active roles	Emotional expression exercises via improvisation	Theatrical improvisations on key scenes	Development of scenes for the final performance
Thursday	Increased Autonomy—Minimal teacher support	Dramatic exploration of story issues (e.g., conflict resolution), dramatized narration	Choral reading and collaborative activities, rehearsals	Synthesis of scenes into a coherent narrative
Friday	Full Student Responsibility	Review of emotions and skills cultivated	Final performance of the theatrical piece and reflection/evaluation	Public presentation (peers, parents, teachers)

**Table 2:** Means, medians, and standard deviations for the dimensions of psychosocial adjustment in the pre- and post-intervention assessments.

Dimension	Stage	Mean	Median	SD	SE
**Social** **competence**	Post-test	52.54	53	9.64	0.36
	Pre-test	51.02	52	10.21	0.38
**Academic** **competence**	Post-test	47.36	46	7.53	0.28
	Pre-test	46.22	45	8.26	0.31
**Emotional** **competence**	Post-test	50.43	51	10.01	0.37
	Pre-test	48.71	49	10.31	0.38
**Behavioral** **problems**	Post-test	50.70	51	9.41	0.35
	Pre-test	53.16	54	8.24	0.31

**Table 3:** Mean differences before and after the intervention in the dimensions of psychosocial adjustment.

Dimensions	t	df	*p*	Mean Diff.	SE Diff.	95% C.Ι.		d	95% C.Ι.	
						LL	UL		LL	UL
**Social** **competence**	5.66	718	<0.001	1.52	0.27	0.99	2.05	0.21	0.14	0.29
**Academic** **competence**	6.3	718	<0.001	1.14	0.18	0.79	1.5	0.24	0.16	0.31
**Emotional** **competence**	6.36	718	<0.001	1.71	0.27	1.19	2.24	0.24	0.16	0.31
**Behavioral** **problems**	−11.74	718	<0.001	−2.46	0.21	−2.87	−2.05	−0.44	−0.51	−0.36

***Note.*** Mean diff. = Mean difference, SE diff. = Standard error of the difference, LL = Lower limit of confidence interval, UL = Upper limit of confidence interval, d = Cohen’s d.

**Table 4:** Reliable Change Index, percentages of change, and ceiling effect across dimensions.

Dimensions	RCI	Improvement %	Stability %	Deterioration %	Ceiling Effect %	Adjusted Improvement %
**Social** **competence**	9.81	11.54	82.06	6.4	11.54	13.04
**Academic** **competence**	5.12	17.11	74.41	8.48	12.66	19.59
**Emotional** **competence**	13.71	5.01	93.05	1.95	11.13	5.64
**Behavioral** **problems**	12.09	4.73	94.71	0.56	10.88	5.31

**Table 5:** Themes, codes and descriptions for qualitative data analysis.

Themes	Codes	Description
**Social** **competence**	Cooperation	The child’s ability to cooperate harmoniously with peers and adults, demonstrating a willingness to participate and respect.
	Assertion	The ability to express one’s needs, thoughts, and emotions in a clear and socially acceptable manner.
**Academic** **competence**	Academic engagement	The ability to meet classroom demands and consistently complete academic tasks.
	Learning motivation	The child’s interest, curiosity, and persistence in learning processes.
**Emotional** **competence**	Self-regulation	The ability to regulate emotional responses, particularly in moments of tension or conflict.
	Empathy	The ability to recognize, understand, and respond to the emotions and needs of others.
	Emotion management	The ability to recognize and process one’s own emotions in a healthy and constructive manner.
	Stress coping	The ability to manage situations that induce stress or pressure using adaptive and effective strategies.
**Behavioral** **problems**	Hyperactivity/Impulsivity	The frequency and intensity of behaviors associated with difficulty concentrating, excessive activity, and impulsive reactions.
	Aggression	Behaviors involving verbal or physical aggression, hostility, or provocation.

## Data Availability

The data presented in this study are available on request from the corresponding author.
